# Being involved in research as a collaborator with experience of a prenatal diagnosis of congenital heart defect in the fetus: a qualitative study

**DOI:** 10.1186/s40900-020-00184-8

**Published:** 2020-03-31

**Authors:** Tommy Carlsson, Ulla Melander Marttala, Elisabet Mattsson

**Affiliations:** 1 Department of Women’s and Children’s Health, Uppsala University, MTC-huset, Dag Hammarskjölds väg 14B, 1 tr, 752 37 Uppsala, Sweden; 2 The Swedish Red Cross University College, Huddinge, Sweden; 3 Department of Scandinavian Languages, Uppsala University, Uppsala, Sweden; 4 Ersta Sköndal Bräcke University College, Stockholm, Sweden

**Keywords:** Congenital heart defects, Community participation, Patient participation, Patient and public involvement, Prenatal diagnosis, Prenatal ultrasonography

## Abstract

**Background:**

An increasing number of research projects are now collaborating with persons who have lived experience of a specific health-related situation, such as a prenatal diagnosis of congenital heart defect. Such collaboration has the potential to provide valuable insights how to plan future studies, but little is known how these persons experience such involvement. The aim was to explore how persons with lived experience of a prenatal diagnosis perceived collaborating in a research project utilizing patient and public involvement to identify relevant research questions and develop suitable interventions.

**Methods:**

Persons with experience of a prenatal diagnosis of congenital heart defect in the fetus were interviewed after their participation in a yearlong collaborative research project (*n* = 9) aiming to explore relevant research questions and develop interventions for expectant parents with a recent prenatal diagnosis. Interviews were analyzed with qualitative content analysis.

**Results:**

Respondents acknowledged altruistic and personal value related to the collaboration. They valued the opportunity to contribute to future research so that the care of persons experiencing a prenatal diagnosis may be improved. Mixed feelings were described related to sharing and reliving experiences. While it had been emotionally difficult to relive a traumatic event, it also served as an opportunity to process experiences and psychologically adapt. Respondents with terminated pregnancies appreciated the possibility to meet peers, since it was difficult to find peers in everyday life and talk about their experiences with others.

**Conclusions:**

Researchers who plan to collaborate with persons who have experience of a prenatal diagnosis should be mindful of the potential associated emotional experiences. The appreciation related to meeting peers calls attention to the need for studies that explore peer support.

## Plain English summary

Many expectant parents attend an ultrasound scan during pregnancy, which can be used to detect fetal anomalies such as congenital heart defects. Involving persons with lived experience to plan and conduct research that aims to improve the care for expectant parents faced with this diagnosis could be highly valuable. The purpose of this study was to explore how persons with lived experience of a prenatal diagnosis of congenital heart defect in the fetus perceived collaborating with researchers in a preceding research project. Nine persons were interviewed after the conclusion of this previous research project. They collaborated in two separate groups, consisting of parents of a living child with congenital heart defects or persons who terminated the pregnancy after the diagnosis. The interviews revealed that there were altruistic and personal values related to participating in the project. They valued the opportunity to contribute to future research so that the care may be improved further. However, mixed feelings were described related to sharing and reliving difficult experiences. While it had been emotional to relive a traumatic event, it also served as an opportunity to process experiences and adapt to their new situation. Those with terminated pregnancies appreciated the possibility to meet others with similar experiences. The conclusion of the study is that researchers need to be mindful of emotional experiences when planning to collaborate with persons who have experience of a prenatal diagnosis of congenital heart defect in the fetus. More research that investigates peer support among those who terminate a pregnancy is needed.

## Background

### Introduction

Research acknowledges a mismatch between the focus of clinical studies and the interests of the public [1, 2]. To bridge this gap, measures are needed to make research in line with the interests and demands of service users [3]. Patient and public involvement (PPI) is a potential way to approach this issue, defined as “research being carried out ‘with’ or ‘by’ members of the public rather than ‘to’, ‘about’ or ‘for’ them” [4]. PPI can be performed either as consultations with intended service users, collaboration with persons who have lived experience, or as completely user-controlled research [5]. It has garnered increased attention and is supported by many policies, funders and organizations that stress its importance [6–8]. Studies report that PPI is associated with enhanced quality and appropriateness of research [9], better service planning and development, higher degrees of consumer-oriented information, and enhanced dissemination of such information [10]. However, less is known about the personal implications and meaning for those who collaborate in these projects.

Worldwide, expectant parents are offered obstetric ultrasound examination in the second trimester of pregnancy to monitor the pregnancy and assess the fetal anatomy. The introduction of these examinations has resulted in an increased number of prenatally diagnosed fetal anomalies, the most common being congenital heart defects [11]. Most expectant parents accept the offer to undergo the ultrasound examination [12]. However, many lack knowledge about the medical purposes and the possible consequences of the examination [13] and experience psychological distress when presented with a fetal diagnosis [14]. Depending on state laws, expectant parents may have the option to terminate the pregnancy. The decision-making to continue or terminate the pregnancy involves ethical, existential and medical aspects needed to be considered in order to reach an informed decision [15, 16]. It is of great importance that expectant parents are offered sufficient information and psychosocial support following the diagnosis [17]. Although PPI research in the field of prenatal diagnosis has the potential to result in the identification and development of relevant interventions, very little is currently known about how expectant parents experience being involved in research as collaborators.

## Methods

### Aim

The aim was to explore how persons with lived experience of a prenatal diagnosis perceived collaborating in a research project utilizing patient and public involvement to identify relevant research questions and develop suitable interventions.

### Design

This was a qualitative interview study. The findings are reported in line with the Guidance for Reporting Involvement of Patients and the Public (GRIPP2) checklist [18] (Additional file 1).

### Study context

In Sweden, all expectant parents are offered an obstetric ultrasound in the second trimester of pregnancy and the large majority accept the offer to undergo the examination [12]. When a congenital heart defect is suspected, expectant parents are referred to a specialist fetal cardiologist for further consultation. Following confirmation of a fetal heart defect, expectant parents are presented with the option to continue or terminate the pregnancy. When pregnancies are continued, expectant parents are continually followed-up according to the routines at the units for fetal cardiology and fetal medicine. In Sweden, induced abortion is available upon request until 18 completed weeks of gestation and in later gestations after approval from the National Board of Health and Welfare. Typically, induced abortions after 12 completed gestational weeks are performed as medical abortions and vaginal expulsion of the fetus.

### Presentation of the preceding collaborative research project

We conducted a research project in which we collaborated with persons who had lived experience of a prenatal diagnosis of congenital heart defect in the fetus. The project aimed to explore relevant research questions and develop interventions that offered informational and emotional support to expectant parents presented with a diagnosis. The project applied an approach according to the multidimensional framework presented by Oliver et al. [5], and positioned itself as consultative in the initial phases and collaborative in the later phases. Consultation was defined as asking the persons with experience of prenatal diagnosis for their views as means to help guide and inform further research, while collaboration was defined as an ongoing partnership between researchers and the persons with experience of prenatal diagnosis. Two groups were formed that had consecutive meetings during the yearlong project: one consisting of parents of living children prenatally diagnosed with a congenital heart disease (*n* = 5) and another group consisting of persons who terminated the pregnancy following the diagnosis (*n* = 5). Six and seven meetings were held with each group, respectively. For a more detailed presentation of the project and the collaborators, please see Additional file 2.

There are many terms that could be used for persons who are involved in research that utilizes PPI. The term research collaborator was chosen for this study and is used in this article for the persons that were involved in our preceding research project. We consider collaborators the term that most clearly reflects the co-operative approach of our project and because many other terms are not appropriate for persons with experience of a prenatal diagnosis. We acknowledge that other terms may be more common or recognized in the field of PPI research, such as service users, patients and members of the public.

### Data collection

After the conclusion of the research project, the 10 collaborators with experience of a prenatal diagnosis were asked to participate in an individual follow-up telephone interview about their perspectives and experiences related to collaborating in the research project. Of the total 10 collaborators, one mother in the group of parents with living children declined participation. All others accepted and were interviewed (*n* = 9). A semi-structured interview guide was used during the interviews (Table 1). The collaborators had no previous contact amongst each other, except for two persons in each group who were couples. A research nurse who had not been an active member of the research team before performing the interviews conducted the interviews. The mean length of the interviews was 32 min (range = 17–42). The interviews were audio-recorded and transcribed verbatim.

**Table 1:** Semi-structured interview guide

Main question	Follow-up questions
Can you describe how it was for you to work with the group?	How was it to share your experiences together with the other members in the group?
	What did it mean for you to talk about your experiences together with the other members?
	What kind of feelings and thoughts did you have when you talked about your experiences with the other members?
	How open with your experiences, feelings and thoughts did you feel that you could be when talking to the other members?
	What positive aspects related to participating in the project were there for you?
	What negative aspects related to participating in the project were there for you?
	How do you feel today about the fact that you decided to participate in the project?
How do you feel about participating in planning of future research?	How did you experience the method of meeting a group consisting of others with similar lived experience as you have, with the purpose to plan future research?
	Is there any other method besides group meetings that you would have preferred?
	How do you feel that the group collaborated?
	What influence do you feel that you had on future research?
What perspectives do you have concerning when researchers collaborate with persons who have lived experience to plan future research?	What benefits do you think there is when researchers collaborate with persons who have lived experience?
	What problems do you think result when researchers collaborate with persons who have lived experience?

### Data analysis

The transcripts were analyzed with qualitative content analysis, inspired by the outline presented by Graneheim and Lundman [19]. All transcripts were read repeatedly to gain an understanding of the overall content. Thereafter, each transcript was scrutinized to identify meaning units, defined as segments of the text that spoke of the same central meaning, related to each other regarding their content and context. The identified meaning units were structured into themes illustrating the experiences conveyed in the interviews. The first author was responsible for the primary analysis, a specialist nurse-midwife with experience of content analyses and the researcher who was the moderator for the meetings. To explore the interviews from several perspectives, the second and last authors read all interview transcripts and reviewed the thematization. The analysis was a flexible and iterative process that continued until all authors felt that the interviews were adequately represented in the thematization and results. The second author is a linguistic researcher and associate professor who attended and collaborated in all meetings. The last author is a specialist nurse-midwife and professor who attended one workshop among the parents of children with congenital heart defects. The interviews were conducted, transcribed and analyzed in Swedish. The quotes used in the manuscript were translated from Swedish to English and checked by a professional proofreading agency.

### Ethical considerations

The investigation conforms with the principles outlined in the Declaration of Helsinki. The study was approved by the Ethical Board in Uppsala, Sweden (approval number 2014/504/1). Oral and written information about the study was provided to all respondents and written informed consent was collected before data collection.

## Results

Two overarching themes were identified. *Finding altruistic value in trying to improve the future care and contribution to future research by being an active collaborator in the research team* portrays the perspectives and experienced value related to working in a project that aims to plan future studies and improve the care of expectant parents. *Experiencing mixed feelings of personal benefits and simultaneously reliving traumatic experiences when meeting peers* portrays the aspects related to finding personal benefits and psychological adaptation by participating in group meetings, but simultaneously experiencing stressful emotional difficulties when reliving previous experiences.

### Finding altruistic value in trying to improve the future care and contribute to future research by being an active collaborator in the research team

Having the opportunity to contribute to future research was highly appreciated, and respondents felt like they were listened to and taken seriously when collaborating in the project. Having a potential impact on future studies was a positive experience associated with pride, gratefulness and hopefulness. The respondents felt like active and influential collaborators during the course of the project. They called attention to the value of using PPI when developing relevant and high-quality information for expectant parents, as information could be produced according to the preferences and needs of the intended consumers.

*I do feel that the actual decision that we reached, that a website is the best format, that decision was made by the group. I consider the collaborating researchers as part of the group.* (Male 1 with terminated pregnancy).

Respondents valued that they were invited to collaborate in the project. They considered PPI a tool with great potential to offer researchers valuable insights, so that they may conduct studies that correspond to the needs of expectant parents. From their perspectives, research projects that do not use PPI are at risk of being overly theoretical and not grounded in the needs of the population. The respondents felt that researchers who collaborate with persons with lived experience can gain new insights which may otherwise be overlooked or disregarded.

*I think it’s necessary to conduct this type of research, where you involve people with this type of background. Otherwise, it’s a great risk that you disregard certain important things, that researchers develop something that does not fully captures the actual problem.* (Father 1, parent of a child prenatally diagnosed with heart defect).

Being an active collaborator in a research project that aimed to improve the care of persons faced with a prenatal diagnosis was highly appreciated. Respondents felt that their contributions had a deeper meaning, since it had the potential to result in an easier and less stressful situation for those presented with a diagnosis. The possibility to further improve the care was a decisive factor that drove them to take part in the project, even if it meant processing stressful emotions. Having contributed to improving the care involved positive feelings with a sense of personal value and pride.

*It’s a sense of personal value to feel that I have [contributed with] information. That I have gone through something that can help others. And that makes it, well, it’s great that I have something to offer.* (Male 2 with terminated pregnancy).

Issues related to PPI were also expressed. Some experienced the scope of the project too narrowly, and thus, felt like it did not allow them to fully express or propose other potential research topics of interest. Another issue involved the constellation of members in the groups, including having the possibility of attending meetings together with their partner, which had not been possible for one respondent because of personal reasons. Others felt that some members were not as active in the discussion as themselves, and thus felt a need to constrain themselves to let others have a chance to take part in the discussions. At times, it had been difficult to fully contribute to the work because some of the activities had a high degree of difficulty and required knowledge about research methodology.

*I was one of those who talked rather much even if I tried to hold back because I didn’t want to talk too much, since I wanted others to also come forward.* (Father 1 of child prenatally diagnosed with heart defect).

### Experiencing mixed feelings of personal benefits and simultaneously reliving traumatic experiences when meeting peers

Respondents described that, on a personal level, they experienced mixed feelings about participating in the project. Participating as a collaborator in research involved dealing with stressful feelings when talking about their experiences related to the prenatal diagnosis, resulting in thoughts and emotions that felt difficult to manage. On the other hand, taking part in the discussions presented an opportunity to talk about and process their experiences, which lead to psychological adaptation. Venting their feelings and experiences together with a group of peers with similar experiences was considered an emotional relief and felt liberating.

*It was also, in some ways, a help to move forward. [...] It felt like a relief, and also in some way, um, mostly I consider that it felt nice, that’s probably really all I can say. Then, of course, sometimes I left [the meetings] and felt sad, but that was not because of the group but rather because it tore up thoughts about it all. [...] It also became a follow-up for what you had experienced because, from the perspective of the healthcare system, it’s like, after the termination you don’t have any kind of contact with the healthcare system at all. So it was mostly like it [the PPI work] allowed longer time, in some way to let it all sink in.* (Female 2 with terminated pregnancy).

Collaborating with others who shared similar experiences presented an opportunity to emotionally connect and bond with peers that understood their experiences. The magnitude in which these peers could understand how it is when being presented with a prenatal diagnosis of fetal anomaly was considered special and on a deeper level compared to friends and relatives.

*It felt nice to... Well, it’s nonetheless others [...] who have similar experiences. So it’s nice to have an opportunity to compare a little, talk about how it all felt. It was other people there who understood the dilemma you go through.* (Mother 3, parent of a child prenatally diagnosed with heart defect).

Respondents with terminated pregnancies pointed out the experienced difficulties finding peers who shared similar experiences. Talking about their experiences was considered a taboo subject not discussed with people around them in their social networks. Thus, meeting other persons with similar experiences gave those involved an opportunity to talk about subjects that they did not talk about in their everyday lives. This was highly valued and seen as a way to emotionally process the traumatic event. The peer-to-peer interaction served as a confirmation that they were not the only ones who decided to terminate the pregnancy and was a way for them to validate their decision.

*I did not have any contact with someone who had experienced the same thing, so it [participating in PPI work] was also in some way a chance to process together with others who had been in the same situation.* (Female 2 with terminated pregnancy).

Taking part in the project was valued because it felt like their perspectives were considered important and mattered to researchers. By contributing to the project, respondents felt listened to and validated by persons who felt genuinely interested to hear about their opinions and experiences. As collaborators in research, they also felt a great trust placed upon them.

*It felt like, even if [the researchers] had not gone through the same thing as us, that it felt very nice. They listened and were very interested, genuinely interested to learn about what we had experienced, what we thought could change, and what we thought you could do to help others in this situation. It felt very welcoming.* (Female 1 with terminated pregnancy).

A need for trust between collaborators was seen as necessary before feeling comfortable to disclose personal perspectives. The longitudinal method was appreciated, as it fostered the possibility to feel more and more comfortable as the project proceeded. Towards the end of the project, respondents had established a personal bond with each other and felt comfortable to speak freely. The fact that the project used face-to-face meetings was valued, as long-distance solutions such as web-based discussions would not have resulted in as honest and openhearted exchanges as the face-to-face meetings did. The respondents also called attention to the importance of well-sized groups of collaborators.

*In the beginning, there was a phase when we got acquainted with one another, when you are supposed to get to know each other and establish trust for one another* [...] *I can imagine that the researchers had a rather difficult time trying to get us going with discussions. But in the end, they had more difficulty getting us quiet so that they could get us to talk about the topics that they wanted us to talk about.* (Male 2 with terminated pregnancy).

## Discussion

The respondents acknowledged both altruistic and personal value related to collaborating in research. They valued the opportunity to contribute to future research and improve care for other parents, but also experienced mixed feelings related to reliving traumatic experiences. While it was emotionally difficult to relive experiences, it simultaneously served as an opportunity to process their experiences and psychologically adapt.

Finding meaning may increase the chances for psychological adaptation following a traumatic event, including after parental bereavement [20]. Personal benefits related to feeling listened to and empowered when contributing to research have been raised [21], which is in line with our findings. Participating in activities that aim to help others is proposed as a way to cope with parental bereavement [20]. Indeed, an identified motivation for patients and members of the public to engage in PPI activities is having the opportunity to improve treatment and help others [22, 23]. According to the findings in this study, similar effects may be found among those with experience of perinatal loss following a prenatal diagnosis. Research indicates that these persons appreciate peer support [24] and communicate peer-to-peer emotional support online [25]. The described positive effects of joint discussions with peers calls attention to the potential positive effects that peer support may have, possibly resulting in alleviated psychological distress. Considering the lack of research that investigates peer support for this population [26], more descriptive and experimental studies are needed.

While participating in discussions about traumatic experiences was stressful, it also presented an opportunity to talk about topics that felt taboo and were not discussed in other social contexts. Many who undergo an abortion withhold information and feel a need to keep their abortion a secret [27]. A study testing a multi-modal intervention to lessen the impact of socio-cultural negative factors and influences when seeking an abortion concluded that the intervention was helpful and emphasized that more studies are needed [28]. The findings of our study strengthen the already articulated need for studies that test interventions that offer emotional and informational support following an induced abortion [15]. Echoing other studies [21, 29], the findings of this study further illustrate the fact that those who are engaged in PPI activities appreciate the opportunity to meet peers and learn through discussions. While this aspect is not one of the primary reasons behind the implementation of PPI, it nevertheless illustrates that PPI may have positive effects on psychological adaptations on a personal level.

The respondents expressed some negative aspects and issues related to PPI. Previous research reports similar findings, including the risk of emotional burden [21]. We acknowledge that researchers need to carefully consider the risks of inducing psychological distress when involving research partners with experience of prenatal diagnosis. In line with current recommendations, researchers should consider establishing relevant guidelines and be prepared to offer psychological support when necessary [4]. Nevertheless, we argue that the articulated benefits that the respondents related to their participation in the project outweighed the negative aspects they expressed.

There are methodological limitations that need to be considered when interpreting the findings. We interviewed nine respondents who collaborated in a preceding yearlong project that utilized face-to-face meetings. This was an exploratory study providing insights into the experiences of persons who are engaged in PPI projects. The findings illustrate aspects that researchers who plan to use PPI in similar contexts and fields may find useful and insightful. Nevertheless, the small sample size needs to be considered when interpreting the findings of this study. We cannot make claims about the transferability to research projects that use other methods. A research nurse who had attended a proportion of the meetings but had not been an active member of the research team performed the interviews. This approach may have promoted the respondents feeling comfortable being honest about the experiences. However, it is still possible that some experiences were left unsaid during the interviews. Furthermore, it is possible that the first author, who was responsible for the primary analysis, was unsuccessful at identifying all aspects brought up during the interviews. To approach the transcripts from other perspectives, all authors read the transcripts and discussed the analysis until they felt that the findings illustrated the data.

## Conclusions

Persons with lived experience of a prenatal diagnosis articulated personal as well as altruistic values and benefits related to collaborating in a research project. Participating in discussions about their experiences following the diagnosis involved the need to relive traumatic events, resulting in emotional stress. On the other hand, the opportunity to talk and bond with peers who shared similar experiences was highly valued and seen as a way to help psychological adaptation. Researchers who plan to collaborate with persons who have lived experience of prenatal diagnosis should be mindful that these methods are associated with highly personal and emotional experiences. The appreciation of group meetings calls attention to the need for studies that explore peer support following a prenatal diagnosis.

## Supplementary information

**Additional file 1:** Guidance for Reporting Involvement of Patients and the Public (GRIPP2) checklist.

**Additional file 2:** Presentation of the preceding research project collaborating with persons who have lived experience of a prenatal diagnosis of congenital heart defect in the fetus.

## Data Availability

The data that support the findings of this study are available on request from the corresponding author (TC). The data are not publicly available due to them containing information that could compromise research participant privacy/consent.

## Abbreviation

PPI: Patient and public involvement
