# Supplementary material for: Being involved in research as a collaborator with experience of a prenatal diagnosis of congenital heart defect in the fetus: a qualitative study
Source: Res Involv Engagem. 2020 Mar 31;6:10. doi: 10.1186/s40900-020-00184-8 (PMC7110790; doi:10.1186/s40900-020-00184-8)
Supplement: Supplementary file 2 — Additional file 2: Presentation of the preceding research project collaborating with persons who have lived experience of a prenatal diagnosis of congenital heart defect in the fetus. [file 40900_2020_184_MOESM2_ESM.pdf]

**Presentation of the preceding research project collaborating with persons who have lived experience of a prenatal diagnosis of congenital heart defect in the fetus**

A PPI research project that aimed to explore and develop interventions offering information and emotional support to expectant parents presented a prenatal diagnosis of congenital heart defect in the fetus was performed between 2015 and 2016 in Sweden. A consultative and collaborative approach was applied where persons who have lived experience of a prenatal diagnosis attended meetings over the course of one year. The work was inspired by the multidimensional framework presented by Oliver et al. [1]. It was consultative in the initial phases and collaborative in the later phases. Collaborators were defined as laypersons with experience of a prenatal diagnosis, asked to return for consecutive meetings during the course of one year. Consultation was defined as asking the collaborators for their views as a means to help guide and inform further research, while collaboration was defined as an ongoing partnership between researchers and the collaborators. The work was iterative and flexible, meaning that the agenda for each meeting was not fixed and decided collaboratively. Various activities were applied depending on the focus (please see the table below). The first and second authors attended all meetings and were the researchers who collaborated with the persons with experience of a prenatal diagnosis.

*Presentation of the preceding research project in which persons with experience of a prenatal diagnosis of a congenital heart defect in the fetus were involved.*

| **Degree of engagement** | **Meeting** | **Aims and purposes** | **PPI activity** |
|---|---|---|---|
| Consultative | First | Gain insights into relevant future research topics[1] | Focus group discussions |
| | Second | Explore the quality of Swedish patient information websites about congenital heart defects[2] | Written assessments of existing websites and focus group discussions |
| | Third | Investigate the quality of, suitability of, and issues with patient information websites about medically induced second-trimester abortions[3] | Written assessments of existing websites |
| Collaborative | Fourth | Critically review and revise information about congenital heart defects, written by specialist health professionals | Workshops |
| | Fifth | Write information about pregnancy termination from the perspectives of persons with lived experience | Workshops |
| | Sixth | Establish a protocol for the methodological design of a relevant research project according to the preferences and views of the research partners | Workshops together with experts in the field of digital interventions |
| | Seventh | Develop and critically revise a mock-up version of a website as a research intervention containing supplemental information about congenital heart defects/induced abortion | Workshops |

[1]Findings reported in Carlsson T, Melander Marttala U, Wadensten B, Bergman G, Mattsson E. Involvement of persons with lived experience of a prenatal diagnosis of congenital heart defect: an explorative study to gain insights into perspectives on future research. Res Involv Engagem. 2016;2:35; [2]Findings reported in Carlsson T, Melander Marttala U, Wadensten B, Bergman G, Axelsson O, Mattsson E. Quality of patient information websites about congenital heart defects: mixed-methods study of perspectives among individuals with experience of a prenatal diagnosis. Interact J Med Res. 2017;6(2):e15; [3]Findings reported in Carlsson T, Axelsson O. Patient information websites about medically induced second-trimester abortions: a descriptive study of quality, suitability, and issues. J Med Internet Res. 2017;19(1):e8.

The collaborators were recruited via two units for fetal cardiology in Sweden. A purposeful recruitment was applied with regard to country of birth, level of education and age. In total, 10 persons with lived experience of a prenatal diagnosis were recruited. They formed two groups in which one consisted of parents of living children with prenatally diagnosed congenital heart defects (n=5) and the other consisting of persons who terminated the pregnancy following the diagnosis (n=5). In both groups, three were females and two were males. Their ages ranged from 23 to 43 years and their highest levels of education were senior high school (n=2) and university/college (n=8). Seven were born in Sweden and three were born elsewhere. Three did not have any born children, four had one born child, and three had more than one born child. The collaborators had no previous contact amongst each other, except for two persons in each group who were couples.

References

1. Oliver SR, Rees RW, Clarke-Jones L, Milne R, Oakley AR, Gabbay J, et al. A multidimensional conceptual framework for analysing public involvement in health services research. Health Expect. 2008;11:72–84.
